# Late-Onset Paralytic Ileus Following Cesarean Section: A Report of a Rare Case

**DOI:** 10.7759/cureus.81954

**Published:** 2025-04-09

**Authors:** Andreas Dimopoulos, Alexandros Trompoukis, Dimitrios Christakopoulos, Spyridon Kavrochorianos, Eleni Tsiampa

**Affiliations:** 1 Second Department of Obstetrics and Gynecology, Elena Venizelou General and Maternity Hospital, Athens, GRC

**Keywords:** cesarean section, gastrointestinal paralysis, late-onset ileus, paralytic ileus, postoperative complication

## Abstract

Paralytic ileus is a potential complication following cesarean section, though its delayed onset is uncommon and may present diagnostic challenges.

We report a rare case of a 45-year-old woman who developed acute gastrointestinal paralysis 13 days after an uncomplicated elective cesarean section. The patient initially recovered well postoperatively, passing flatus within 24 hours and stool by the third day. However, nearly two weeks after discharge, she presented with abdominal distension, nausea, vomiting, fever, and an inability to pass flatus or stool. Laboratory tests revealed elevated inflammatory markers, and imaging showed gastrointestinal dilation without evidence of mechanical obstruction. Conservative management, including nasogastric decompression, intravenous fluids, and parenteral nutrition, led to a complete resolution without the need for surgical intervention.

Although this condition typically occurs in the early postoperative period, its delayed presentation highlights the need for clinical awareness in postpartum patients with persistent gastrointestinal symptoms. Early recognition and appropriate management are essential for ensuring favorable patient outcomes.

## Introduction

Paralytic or adynamic ileus is a clinical condition that complicates many surgical procedures. The incidence of paralytic ileus varies depending on the type of surgical procedure. It has been reported in 10-20% of cases after a cesarean section [1]. The majority of women who undergo cesarean section regain bowel function, passing flatus within 24 hours and stool within 72 hours postoperatively. Hypoactive bowel function due to paralytic ileus (loss of peristalsis) may occur after abdomino-pelvic surgery. Paralytic ileus usually presents with abdominal distension, nausea, intolerance to oral intake, and an inability to pass flatus or stool that persists for more than three to five days postoperatively. For the diagnosis of paralytic ileus, mechanical obstruction must first be excluded [2].

Little is known about the exact mechanism of adynamic ileus, although it is most likely related to excessive parasympathetic suppression or overstimulation of the sympathetic system, both of which regulate intestinal motility. Various causes have been implicated in the development of paralytic ileus, such as manipulation of the intestinal loops during surgery, intraoperative use of irritating substances, and the release of inflammatory mediators - all of which may dysregulate the intestinal neuronal pathways. Other known risk factors for postoperative paralytic ileus include advanced age, obesity, perioperative administration of opioids, excessive crystalloid use, blood transfusion, and prolonged surgery [3]. According to a six-year observational study, paralytic ileus was also correlated with significant peripartum hemorrhage (>2,000 mL) [4]. Given the potential complications associated with paralytic ileus, timely diagnosis and appropriate management are crucial for improving patient outcomes.

In this report, we present a rare case of a 45-year-old woman who developed acute gastrointestinal paralysis (adynamic ileus) 13 days after an uneventful cesarean section. This delayed presentation is highly unusual and highlights the importance of considering paralytic ileus as a differential diagnosis in post-cesarean patients with gastrointestinal symptoms, even beyond the typical postoperative period.

## Case presentation

A 45-year-old female from Greece presented to the Emergency Department (ED) of our hospital at 38 weeks of gestation, Gravida 1 Para 0 (G1P0). An elective cesarean section was performed due to breech presentation. A low transverse incision was made under spinal anesthesia, and a healthy male newborn weighing 3,230 g, with an excellent Apgar score, was delivered. No adhesions were detected. Her postpartum course was uneventful, with early ambulation, normal decidual sloughing, passage of flatus within the first 24 hours, and bowel movement by the third post-cesarean day. On the fourth postoperative day, she was discharged from the hospital with a prescription for simple analgesics and clinical advice for follow-up.

**Figure 1:**
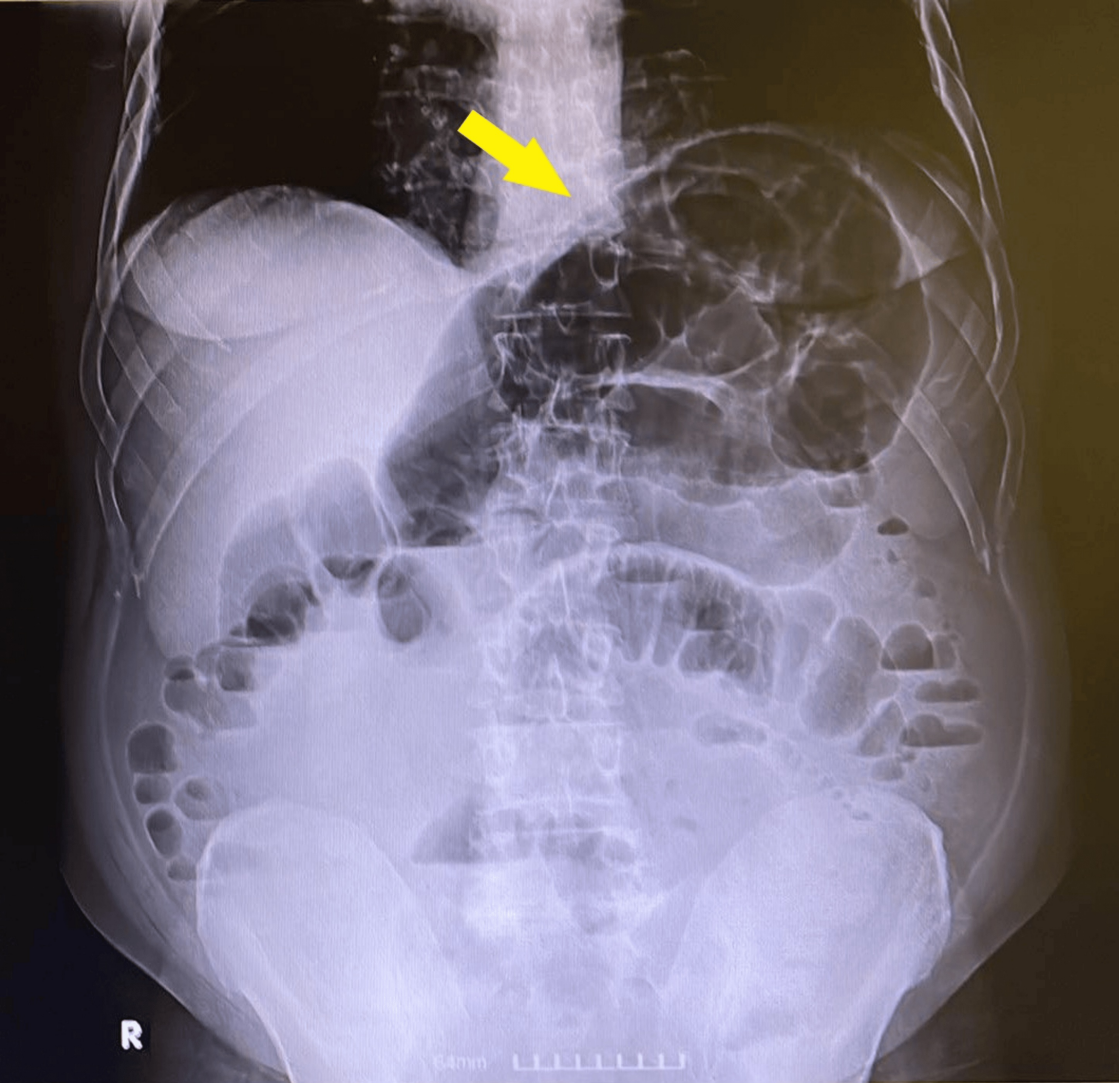
Abdominal X-ray showing signs of diffuse dilation of the small intestine, without a clear transition point (yellow arrow).

**Figure 2:**
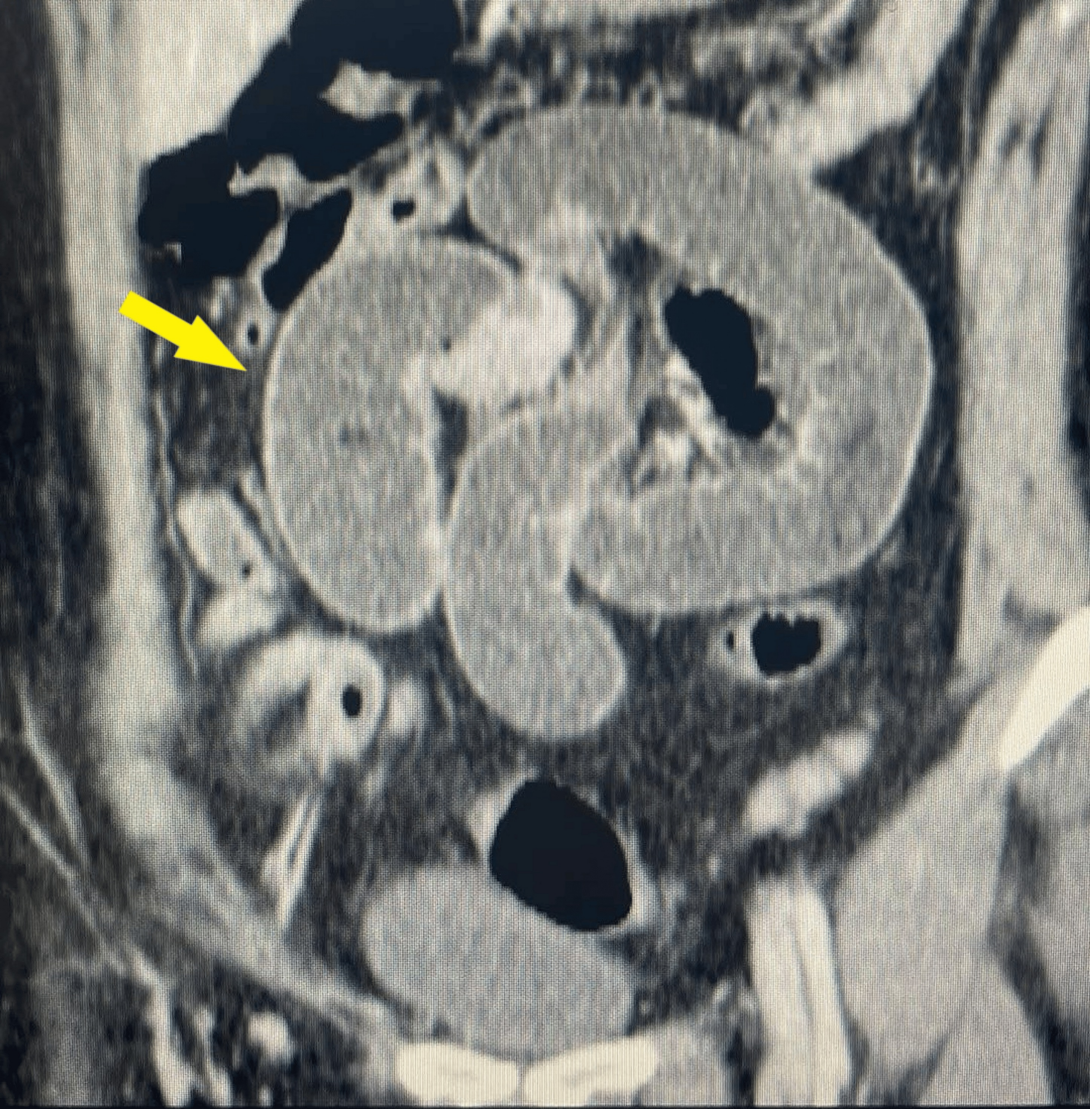
CT scan showing dilated loops of the the small intestine (yellow arrow).

Thirteen days postoperatively, the woman presented to the ED with a two-day history of stool and flatus retention, abdominal bloating, marked fatigue, nausea, vomiting, and fever up to 38.5°C without chills. She did not report abdominal or back pain or dysuria, while her vaginal discharge during the puerperium was normal. However, she exhibited marked agitation. Her past surgical history included laparoscopic ablation of endometriotic lesions. Her family history was unremarkable, and her medication history consisted only of multivitamins during pregnancy and the puerperium. On admission, she reported nausea and episodes of vomiting. Regarding her vital signs, she was febrile with a temperature of 38°C but hemodynamically stable (blood pressure: 122/79 mmHg, heart rate: 75 bpm). Her respiratory rate was 16 breaths/min. Physical examination revealed mild abdominal tenderness and absent intestinal sounds upon auscultation. Breast examination was unremarkable, and the C-section wound showed no signs of infection. Initial laboratory evaluation showed neutrophilic leukocytosis (white blood cells: 16,000/μL; neutrophils: 88%; platelets: 800 × 10^3^/L; hematocrit: 32%; hemoglobin: 10.5 g/dL; C-reactive protein (CRP): 225 mg/L), while liver and kidney function tests were normal. A plain abdominal X-ray revealed gastrointestinal dilation, predominantly involving the stomach (gastroparesis) and small intestine (Figure 1). An abdominal ultrasound scan showed a normal liver, gallbladder, bile ducts, kidneys, and urinary tract, but severe bowel dilation. The uterus and Douglas space appeared slightly enlarged but without any pathological features. A contrast-enhanced abdominal computed tomography (CT) scan showed no free air intraperitoneally (indicating no perforation) and dilation of the stomach and small intestine without other pathological features or obstructive adhesions (Figure 2). There was no evidence of bowel obstruction. An abdominal or pelvic infection was initially considered in the differential diagnosis, given the patient's fever, leukocytosis, and elevated CRP. However, the absence of localized abdominal tenderness, rebound, or guarding, as well as the lack of abnormal findings on imaging (such as intra-abdominal fluid collections, abscess, or uterine pathology), argued against a surgical or gynecologic infection. Furthermore, the cesarean section wound was intact with no signs of infection, and the lochia was normal. These findings, in conjunction with the imaging results showing diffuse bowel dilation without a clear transition point, were more consistent with a diagnosis of postoperative paralytic ileus. Given the above findings, the patient was readmitted to the hospital with suspected postoperative paralytic ileus. A nasogastric tube (Levin 18G) was inserted for stomach and bowel decompression, immediately yielding three liters of gastric fluid. The patient was placed on fasting while intravenous fluids and antibiotics were initiated.

**Figure 3:**
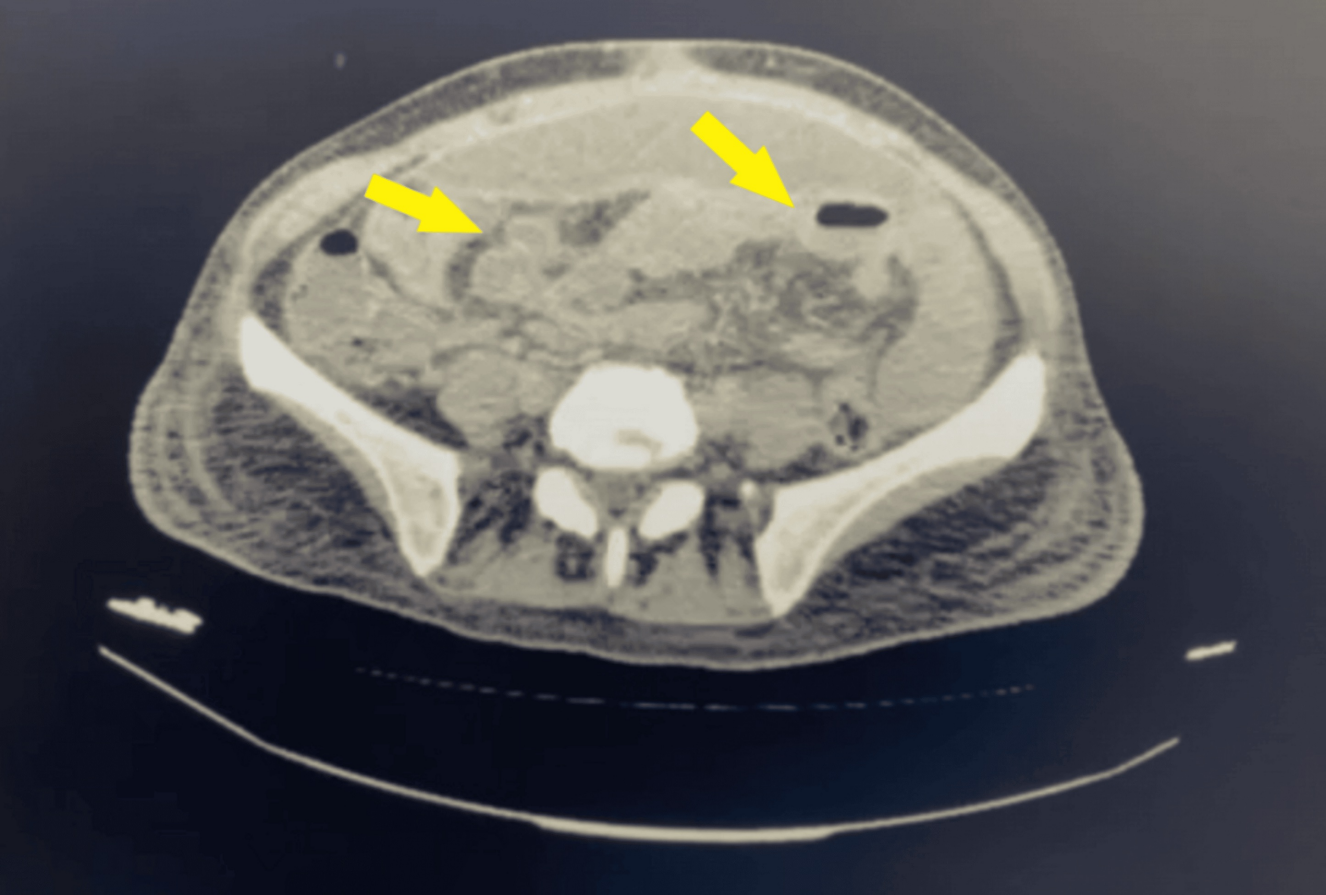
CT scan performed after the initial conservative treatment revealed an improvement in small intestine dilation (yellow arrows).

On the fifth day of hospitalization, parenteral nutrition was initiated. Subsequently, a gastroscopy was performed, revealing mild gastritis. A second CT scan was requested, which showed mild clinical improvement compared to the initial scan (Figure 3). The decision to continue conservative treatment was made jointly by general surgeons and gastroenterologists. The nasogastric tube was removed, and oral Gastrografin was administered. The following day, bowel sounds were auscultated, and a marked improvement in laboratory test results was observed (white blood cells: 8,200/μL; neutrophils: 65%; hemoglobin: 12 g/dL; hematocrit: 35%; platelets: 550 × 10^3^/L; CRP: 14 mg/L) (Table 1). On the ninth day of hospitalization, oral hydration was initiated, and two days later, oral food intake was allowed. The patient tolerated oral nutrition well, and on the 12th day, parenteral nutrition was discontinued. All symptoms resolved, the patient reported normal bowel movements, and laboratory tests returned to normal. Both clinical and laboratory resolution of ileus was confirmed without the need for surgical intervention. After 20 days of hospitalization, the patient was discharged with clinical advice. No signs of recurrence were observed during a six-month follow-up period.

**Table 1:** The patient's laboratory findings.

Laboratory Findings	Patient's Results (Hospitalisation Day 1)	Patient's Results (Hospitalisation Day 6)	Normal Values
White Blood Cell (WBC) (x10³/µL)	16	8.2	4.9-10.8
Neutrophils (%)	88	65	40-75
Hemoglobin (g/dL)	10.5	12	11.8-17.8
Hematocrit (%)	32	37	37.7-47.9
Platelet Count (x10³/µL)	800	550	150-350
C-reactive Protein (CRP) (mg/L)	225	14	<5

## Discussion

Paralytic ileus, though rare, remains a possible complication after cesarean section. Its insidious clinical manifestations can pose a diagnostic challenge for physicians and delay the patient’s recovery. According to a retrospective cohort study, its incidence among 23,486 women was 0.6% [3]. Paralytic ileus has been associated with various symptoms and complications, including nausea and vomiting, dehydration, pulmonary aspiration, and atelectasis. Consequently, it may lead to an increased need for painkillers, delayed patient ambulation, and prolonged hospital stay.

Several measures can be implemented to reduce the risk of paralytic ileus, including minimizing blood loss and operative time, maintaining hemodynamic stability, avoiding opioid use for postoperative pain control, and encouraging early ambulation after surgery. During pregnancy, adopting healthier dietary habits to prevent severe constipation may also help reduce the occurrence of postoperative paralytic ileus. If ileus develops, various pharmacological and non-pharmacological approaches have been described, such as gum chewing, opioid avoidance, correction of electrolyte imbalances, enemas and laxatives, nasogastric tube decompression, and medications like alvimopan, neostigmine, and metoclopramide [4].

The existing literature contains limited reports of functional bowel obstruction following cesarean section, with most cases involving acute colonic pseudo-obstruction affecting the cecum and ascending colon (Ogilvie’s syndrome) [5]. We identified only three articles referring to paralytic ileus after cesarean section [1,4]. In all reported cases, the clinical signs and symptoms of paralytic ileus appeared within the first five postoperative days. To our knowledge, our case is the only one with such a late onset - 13 days postoperatively - as well as the first to describe severe gastroparesis occurring alongside adynamic ileus.

## Conclusions

Delayed bowel function recovery is a common occurrence after cesarean section and can occasionally lead to complications such as paralytic ileus. Post-cesarean patients presenting with significant abdominal symptoms, including pain, distension, and signs of bowel obstruction or paresis, should be promptly evaluated to prevent maternal morbidity and mortality. Obstetricians must maintain a high index of suspicion for paralytic ileus and Ogilvie’s syndrome in such cases, even in the absence of overt colonic obstruction. The possibility of an underlying abdominal infection must be excluded prior to establishing a diagnosis of paralytic ileus. Although paralytic ileus typically presents within the first few postoperative days, rare cases, such as the one described here, may exhibit a delayed onset. This case highlights the importance of recognizing late-onset paralytic ileus as a potential postoperative complication. Its presentation more than 10 days after delivery, despite an initially uneventful recovery and discharge, underscores the need for clinicians to remain vigilant for gastrointestinal complications beyond the immediate postpartum period. Conservative management, including bowel rest, nasogastric decompression, and supportive care, can lead to full recovery without the need for surgical intervention. Awareness of this condition and early intervention are crucial for improving patient outcomes.

The case described here is one of the few reported instances of severe "late-onset" paralytic ileus during the postoperative period. It involves a 45-year-old woman who presented to the ED with adynamic ileus 13 days after an uneventful cesarean section. No evidence of bowel obstruction was found, and conservative management led to the complete resolution of symptoms.
